# Pathogenicity of Highly Pathogenic Avian Influenza A/H5Nx Viruses in Avian and Murine Models

**DOI:** 10.3390/pathogens14020149

**Published:** 2025-02-04

**Authors:** Sara H. Mahmoud, Marwa S. Khattab, Nahed Yehia, Ali Zanaty, Abd El Sattar Arafa, Ahmed A. Khalil

**Affiliations:** 1 Center of Scientific Excellence for Influenza Viruses (CSEIV), National Research Centre (NRC), Dokki, Giza 12622, Egypt; sarahussein9@yahoo.com; 2 Texas Biomedical Research Institute, San Antonio, TX 78245-0549, USA; 3 Pathology Department, Faculty of Veterinary Medicine, Cairo University, Giza 12211, Egypt; 4 Reference Laboratory for Veterinary Quality Control on Poultry Production, Animal Health Research Institute, Agricultural Research Center, Dokki, Giza 12618, Egypt; 5 Veterinary Serum and Vaccine Research Institute (VSVRI), Agriculture Research Center (ARC), Cairo 11381, Egypt

**Keywords:** avian influenza viruses, H5Nx, pathogenicity, transmissibility

## Abstract

The evolution and adaptation of highly pathogenic avian influenza (HPAI) viruses pose ongoing challenges for animal and public health. We investigated the pathogenic characteristics of the newly emerged H5N1/2022 and H5N8/2022 of clade 2.3.4.4b compared to the previously circulating H5N1/2016 of clade 2.2.1.2 in Egypt using both avian and murine models. All strains demonstrated a 100% mortality in chickens after intranasal inoculation (10^6^ EID50), while the H5N8/2022 strain showing significantly higher viral shedding (8.34 ± 0.55 log10 EID50). Contact transmission rates varied between strains (50% for the 2.3.4.4b clade and 100% for the 2.2.1.2 clade). In the mouse model, H5N1/2016 infection resulted in an 80% mortality rate with significant weight loss and virus replication in organs. In contrast, H5N8/2022 and H5N1/2022 had 60% and 40% mortality rates, respectively. An histopathological analysis revealed pronounced lesions in the tissues of the infected mice, with the most severe lesions found in the H5N1/2016 group. These findings suggest the decreased pathogenicity of the newer H5Nx strains in mammalian models, emphasizing the need for continued surveillance and adaptive control strategies.

## 1. Introduction

Highly pathogenic avian influenza (HPAI) viruses, in particularly the H5N1 and H5N8 subtypes, remain major threats to global poultry populations, wildlife, and public health [1,2]. The virus demonstrates a remarkable ability for rapid geographic dissemination, causing severe economic losses, while raising concerns about potential zoonotic transmission [3,4]. This evolutionary process has led to the establishment of separate clades for the HPAI H5N1 and H5N8 subtypes, which further supports their intrinsic dynamic nature in the emergence of strains with an altered pathogenicity, replication efficiency, and transmissibility [5,6].

In Egypt, HPAI outbreaks have devastated poultry populations for the past two decades, with economic losses exceeding USD 2.5 billion since 2006 [7,8]. Recent surveillance data from 2022–2023 shows alarm, with 431 confirmed outbreaks distributed through 23 governorates affecting approximately 2.8 million birds [9]. The poultry sector in Egypt has presented certain peculiarities, with a dense population and diverse farming systems that provide ideal conditions for virus evolution and spread [10]. Current data show the emergence of novel clade 2.3.4.4b strains, coinciding with an increased frequency and severity of outbreaks [11].

The simultaneous circulation of HPAI viruses, especially the H5N1/2022 and H5N8/2022 of clade 2.3.4.4b, alongside the historically prevalent H5N1/2016 of clade 2.2.1.2, presents complex challenges in the Egyptian poultry ecosystem [12,13]. Those co-circulating strains pose many important questions related to their comparative behavior, effect, and evolutionary trajectory [14].

In general, the clade 2.3.4.4b virus incursion has indicated major shifts in the ecology of HPAI viruses, because those newly emerged viruses have displayed properties quite distinct from the clade 2.2.1.2 viruses circulating formerly [15,16].

As these HPAI strains are under selective pressure in the dynamic Egyptian poultry environment, the genetic constitution of these strains might give important information concerning their adaptability and outbreak potential [17,18].

Evolution through genetic changes can appreciably alter the host behavior, transmissibility, and host range of the strain [19]. Active surveillance shows the co-circulation of multiple viral genotypes that could create conditions for continued evolution by reassortment and adaptation [20,21].

Our study addresses critical knowledge gaps on the comparative pathobiology of these co-circulating HPAI strains. We provide novel insights into virus evolution and its implications for disease control through the examination of recent strains (H5N1/2022 and H5N8/2022 of clade 2.3.4.4b) and a historical strain (H5N1/2016, clade 2.2.1.2) [22,23].

This is the first full comparison of these strains in avian and mammalian models, and it has provided very important data usable in risk assessment and for the development of control strategies [24].

The focus on H5N1/2022 is due to its recent establishment as a dominant subtype in avian populations. A comparative study with the co-circulating H5N8/2022 and the previously dominant H5N1/2016 may elucidate differences in pathogenicity, growth dynamics, and transmission potential [25,26].

The aim of this work is to provide fundamental insights into the mechanisms of avian influenza virus evolution and its implications for animal and public health through the thorough characterization of these viral strains [27,28].

## 2. Materials and Methods

### 2.1. Virus Strains

The following HPAI virus strains were used in this study (Table 1):

The key molecular features included the presence of multiple basic amino acids at the HA cleavage site characteristic of HPAI viruses. All viral isolates were propagated in specific pathogen-free (SPF) embryonated chicken eggs to prepare working stocks. Virus titers were determined using the standard method for calculating the 50% egg infective dose (EID50) according to Reed and Muench [29].

### 2.2. Animal Studies

All experiments were conducted in the BSL3 containment facility at the Veterinary Serum and Vaccine Research Institute (VSVRI), following institutional biosafety guidelines. The study compared the pathogenicity, replication efficiency, and transmissibility of three HPAI viruses using both chicken and mouse models. Animal experiments were approved by the Institutional Animal Care and Use Committee (Approval code: ARC, VSVRI, 01,25).

#### 2.2.1. Chickens Experimental Design

Specific pathogen-free (SPF) chickens (*n* = 52, 4 weeks old) were obtained from Koum Oshiem SPF Chicken Farm (Fayoum, Egypt). The chickens were randomly divided into four experimental groups (*n* = 10 per group) for direct inoculation: G1 received Strain H5N1/2022 (2.3.4.4b), G2 received Strain H5N8/2022 (2.3.4.4b), G3 received Strain H5N1/2016 (2.2.1.2), and G4 served as the PBS control. For the transmission studies, three additional groups of contact chickens (*n* = 4 each) were introduced 24 h post-infection to G1, G2, and G3, designated as G1\, G2\, and G3\, respectively. Each chicken in the inoculated groups received 10^6^ EID50 of the respective virus intranasally in a 0.1 mL volume. Clinical monitoring: Daily for 14 days. Oropharyngeal and cloacal swabs were collected on days 2, 4, 6, and 8 post-infection. Tissue samples from the trachea and lungs were collected on days 3 and 5 post-infection for viral load quantification and histopathological examination.

#### 2.2.2. Mouse Experimental Design

Female Black 6 mice (*n* = 26, 6-weeks-old) were obtained from the Animal House Colony of the National Research Center (NRC, Egypt). Mice were randomly assigned to four groups (*n* = 5 per group): G1 (H5N1/2022), G2 (H5N8/2022), G3 (H5N1/2016), and G4 (PBS control). Following CO2 anesthesia, the mice were inoculated intranasally with 10^6^ EID50 in a 100 μL volume.

Body weight and clinical signs were monitored daily for 14 days. Neurological assessment was performed twice daily using a standardized scoring system. Tissue samples from lungs, liver, and spleen were collected on days 3 and 5 post-infection for viral load determination and histopathological analysis.

All experimental procedures were conducted in accordance with institutional animal care guidelines under approved protocols.

#### 2.2.3. Sample Size Calculation

Sample sizes were determined using G*Power 3.1 software. For the chicken experiments, based on previous studies showing a 100% mortality in HPAI infections, a sample size of 10 birds per group was calculated to achieve a 90% power to detect a hazard ratio of 2.0 using a two-sided log-rank test with α = 0.05. For the transmission studies, 4 contact birds per group were determined sufficient to detect 50% transmission with an 80% power.

For the mouse experiments, a sample size of 5 mice per group was calculated to provide an 80% power to detect a 20% difference in survival rates between groups using the chi-square test with α = 0.05, based on anticipated mortality rates from pilot studies. These calculations incorporated an expected 10% attrition rate.

#### 2.2.4. Randomization and Control of Confounders

Animals were randomized to experimental groups using a computer-generated random number sequence (Research Randomizer v4.0). To minimize confounders, treatment groups were balanced across cages/rooms, sample collection was performed in a randomized order, and cage positions were rotated daily. All measurements and sample collections were conducted at consistent times of day following standardized protocols.

#### 2.2.5. Blinding Procedures

Personnel conducting behavioral assessments, histopathological analyses, and data analysis were blinded to group allocation. Animal care staff and personnel performing viral inoculations were necessarily aware of group assignments to ensure the proper handling of infectious materials. A separate researcher not involved in outcome assessments performed the group allocation.

### 2.3. Histopathological Examination

Tissue specimens from the trachea and lung of chickens, and also the lung, liver, and spleen from mice, were fixed in 10% neutral buffered formalin. Tissues were processed by an ascending concentration of ethanol and xylene and embedded in paraffin. Tissue sections (4 µm thick) were made using a rotary microtome and stained by hematoxylin and eosin stain. Light microscopy was used for the examination, and a digital camera was used for photography. The edema, congestion, hemorrhage, degenerative changes, necrosis, and inflammatory cell infiltration were assessed based on a modified scale, in which 1 = none or negligible, 2 = mild, 3 = moderate, 4 = moderate to severe, and 5 = severe [12].

### 2.4. Viral Titration

Sampling Schedule: For chickens, oropharyngeal and cloacal swabs were collected at 2, 4, 6, and 8 days post-infection (dpi), and organ samples (trachea, lungs) were collected at 3 and 5 dpi. For mice, organ samples (lungs, liver, spleen) were collected at 3 dpi and 5 dpi. Swabs and tissue samples were collected and processed according to standardized protocols. The collected swabs were placed in viral transport medium, while 0.1 g of the collected organs underwent homogenization in phosphate-buffered saline using the tissue lyser LT (Qiagen, Hilden, Germany). The samples underwent three cycles of freezing and thawing, followed by centrifugation at 12,000× *g* for 10 min. The supernatant was separated after centrifugation.

RNA extraction was performed using the QIAamp Viral RNA Mini Kit, following the manufacturer’s instructions. RNA quality and quantity were assessed using a Nano Drop TM 2000 spectrophotometer. The reverse transcription and amplification of the H5 gene (segment 7) were conducted using the Quantitect probe reverse transcriptase–polymerase chain reaction kit (Qiagen, Hilden, Germany). The specific primers and TaqMan probe sequences were as follows [15]:

Sep1: 5′-AGATGAGTCTTCTAACCGAGGTCG-3′;

Sep2: 5′-TGCAAAAACATCTTCAAGTCTCTG-3′;

Probe SePRO: [FAM]-TCAGGCCCCCTCAAAGCCGA-[TAMRA].

Each RT-PCR run included a standard curve generated using 10-fold serial dilutions (10^1^ to 10^8^ EID50/mL) of quantified virus stock and no-template controls. Viral loads were determined by the interpolation of CT values from the standard curve and expressed as the log10 EID50 per ml or gm of tissue.

### 2.5. Data Collection and Statistical Analysis

Data were analyzed using GraphPad Prism version 5.0 for Windows (GraphPad Software, San Diego, CA, USA), using the Kaplan–Meier method [13], and SPSS Statistics for Windows, Version 28.0 (IBM Corp., Armonk, NY, USA) [30]. Statistical significance was determined at *p* < 0.05 unless otherwise specified. Prior to analysis, the data distribution was assessed using the Shapiro–Wilk test for normality and Levene’s test for homogeneity of variance [31].

For normally distributed data with homogeneous variance, parametric tests were employed: independent *t*-tests for two-group comparisons and a one-way ANOVA with appropriate post hoc tests for multiple comparisons [32]. For non-normally distributed data, non-parametric alternatives were used: the Mann–Whitney U test (two groups) and Kruskal–Wallis test with Dunn’s post hoc test (multiple groups) [30,33]. When data violated the homogeneity of variance assumption, Welch’s ANOVA with the Games–Howell post-hoc test was applied [34].

Correlations between viral loads and clinical parameters were assessed using Spearman’s rank correlation coefficient, with significance set at *p* < 0.001 [35]. Survival curves were analyzed using a log-rank test for comparing survival rates between virus groups [13].

The results are presented as mean ± standard deviation for normally distributed data, median with interquartile range for non-normal distributions, and percentages for categorical variables [36].

### 2.6. Ethical Considerations

This study was approved by the Institutional Animal Care and Use Committee at the Agricultural Research Center (Approval code: ARC, VSVRI, 01,25). All procedures were performed in accordance with the guidelines of the National Institute of Health (NIH).

All methods are reported in accordance with ARRIVE guidelines [37].

## 3. Results

### 3.1. Pathogenicity of Highly Pathogenic Avian Influenza A/H5Nx Viruses in Avian Species

#### 3.1.1. Morbidity and Mortality Rate of Chickens

All three HPAI virus strains demonstrated a high pathogenicity in chickens following intranasal inoculation with 10^6^ EID50 (Table 2). Clinical signs first appeared at 3 days post-infection (dpi), characterized by severe respiratory distress, lethargy, and reduced feed intake. Mortality patterns varied among groups. H5N1/2022 (G1) exhibited 100% mortality by day 7, with peak deaths (4/10) occurring at 4 dpi. H5N8/2022 (G2) also reached 100% mortality by day 5, showing a more rapid disease progression. H5N1/2016 (G3) demonstrated 100% mortality by day 7, with a similar pattern to G1. The control group (G4): No clinical signs or mortality. In the contact groups, mortality rates were G1\(H5N1/2022 contacts): 50% (2/4) and G2\(H5N8/2022 contacts): 50% (2/4), while G3\(H5N1/2016 contacts): 100% (4/4).

#### 3.1.2. Shedding and Replication Kinetics

**Figure 1:**
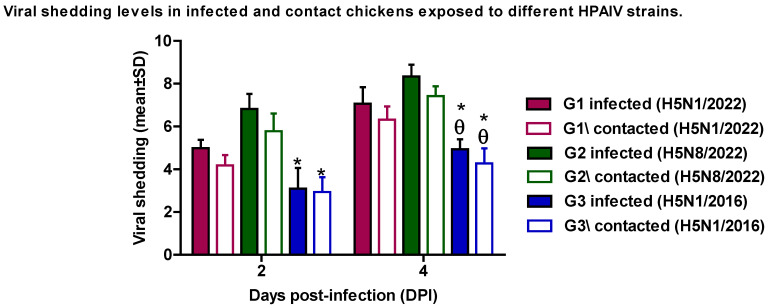
Viral shedding levels (log10 EID50/mL) in infected and contact chickens with different HPAIV strains. Values are presented as mean ±SD. θ: statistically significant compared to corresponding value in group 1 (*p* < 0.05). *: statistically significant compared to corresponding value in group 2 (*p* < 0.05).

Viral shedding was quantified from oropharyngeal and cloacal swabs at 2 and 4 dpi (Figure 1). The Kruskal–Wallis test revealed significant differences in viral shedding patterns between strains (H = 12.34, *p* < 0.05). The H5N8/2022 isolate demonstrated the highest shedding levels (8.34 ± 0.55 log10 EID50/mL at 4 dpi) compared to other H5N1/2022 and H5N1/2016 strains (7.06 ± 0.78 and 4.94 ± 0.46, respectively), which potentially indicated enhanced transmissibility (*p* < 0.05 compared to both 2022 strains).

Contact transmission studies revealed similar strain-dependent patterns. At 4 days post-contact, the H5N8/2022 strain maintained the highest viral shedding levels (7.42 ± 0.46 log10 EID50/mL), followed by H5N1/2022 (6.32 ± 0.62 log10 EID50/mL) and H5N1/2016 (4.27 ± 0.71 log10 EID50/mL). Welch’s ANOVA with the Games–Howell post hoc test demonstrated significant differences between the 2022 strains and the 2016 strain (F = 21.34, *p* < 0.05). These findings suggest the enhanced transmission potential of the newly emerged H5N8/2022 strain compared to contemporaneous and historical H5N1/2016 strains.

#### 3.1.3. Histopathological Findings in Chickens

**Figure 2:**
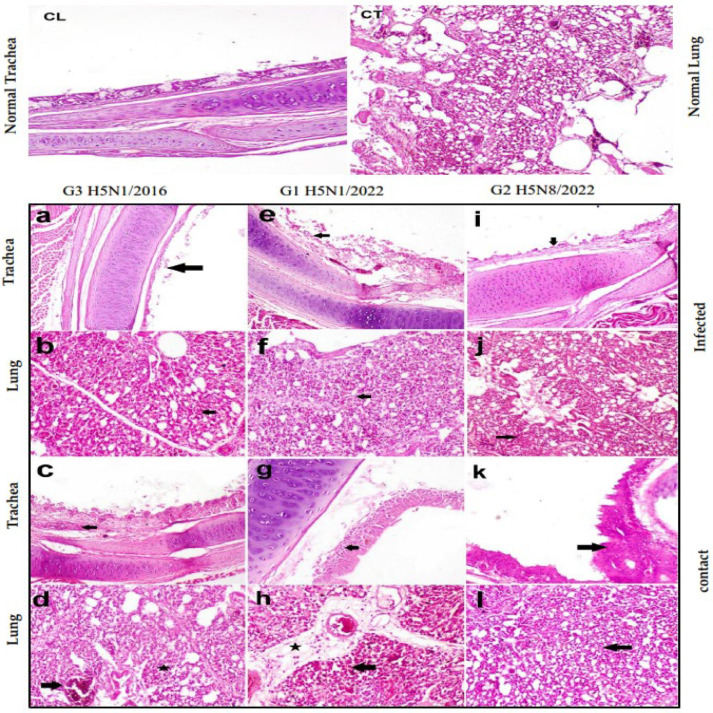
Histopathology of trachea and lungs in different groups. (**CL**) Control lung and (**CT**) control trachea. (**a**) Severe ulcerative tracheitis, (**b**) mild leukocytic infiltration in infected birds of H5N1/2016 group, (**c**) ulceration of lining epithelium of trachea in H5N1/2016 contact birds, (**d**) moderate congestion, edema, and leukocytic infiltration in lungs of H5N1/2016 contact birds, (**e**) severe ulceration, congestion, and leukocytic infiltration in trachea of H5N1/2022 group, (**f**) severe pneumonia in lungs of H5N1/2022 group, (**g**) moderate leukocytic cell infiltration in trachea of contact birds to H5N1/2022, (**h**) severe interstitial edema and moderate leukocytic cells infiltration in lungs of contact birds to H5N1/2022, (**i**) severe diffuse ulceration in trachea of H5N8/2022, (**j**) mild congestion, edema, and leukocytic infiltration in lungs of H5N8/2022 group, (**k**) hyperplasia of lining epithelium and deciliation in trachea of contact birds of H5N8/2022 group, (**l**) mild congestion, edema, and leukocytic infiltration in lungs of contact birds of H5N8/2022 group. Black arrows indicate the following: in trachea—areas of epithelial damage/ulceration; in lung—inflammatory infiltrates and congestion. Star indicate inflammatory cells infiltration in the wall of air capillaries. Scale bars = 50 µm. Hematoxylin and eosin stain (X200).

The tissue examination revealed strain-specific patterns of pathology (Figure 2):

G1 (H5N1/2022) exhibited severe tracheal ulceration, congestion, and leukocytic infiltration, with severe pneumonia (Figure 2e,f). Contact birds displayed moderate tracheal leukocytic infiltration and severe interstitial edema with moderate leukocytic infiltration in lungs (Figure 2g,h). G2 (H5N8/2022) demonstrated severe diffuse tracheal ulceration, with mild pulmonary congestion, edema, and leukocytic infiltration (Figure 2i,j). Contact birds showed epithelial hyperplasia, deciliation, and mild pulmonary congestion with leukocytic infiltration (Figure 2k,l). G3 (H5N1/2016) revealed severe ulceration in the tracheal epithelium with moderate congestion and leukocytic infiltration, alongside mild leukocytic infiltration in lungs (Figure 2a,b). Contact birds showed tracheal epithelial ulceration and moderate pulmonary congestion, edema, and leukocytic infiltration (Figure 2c,d).

**Figure 3:**
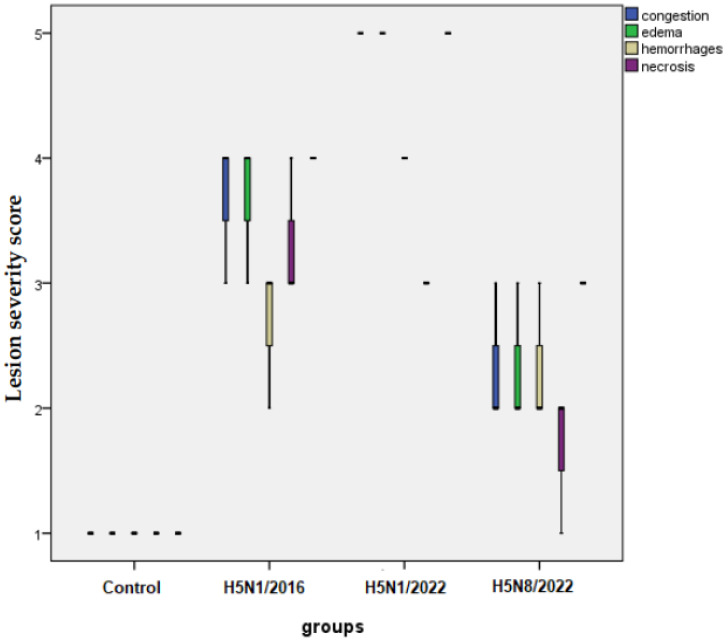
Distribution of histopathological lesion scores in chicken lungs. Boxplots show median, quartiles, and range of scores for each group (*n* = 10 per group). The medians are the thick middle lines. The maximum and minimum values are represented by the thin horizontal lines at the top and bottom. Scoring system: 1 = none/negligible, 2 = mild, 3 = moderate, 4 = moderate to severe, 5 = severe. Parameters scored included edema, congestion, hemorrhage, necrosis, and inflammatory cell infiltration. Data generated using GraphPad Prism V5 software. *p* < 0.05 compared to control group; *p* < 0.05 between indicated groups.

Lesion scoring revealed the highest severity in the H5N1/2022 group, presented in a boxplot (Figure 3), with significant differences in edema, congestion, inflammatory cell infiltration, and tissue necrosis.

All strains caused severe damage to the trachea and lungs, with H5N1/2022 appearing to cause the most severe lesions based on the boxplot of lesion scores

### 3.2. Pathogenicity of HPAI- H5 Viruses in Mice

#### 3.2.1. Pathogenicity in Mice

The pathogenicity of the three HPAI virus strains (H5N1/2022, H5N8/2022, and the previously circulating H5N1/2016) was assessed in twenty-six-weeks mice, which were divided into four groups, with five mice in each group and intranasal inoculation with 100ul of 10^6^ TCID50/0.1ml viral doses. The mice were monitored for clinical signs and mortality over a specific period and for viral replication in mice organs via a histopathology for lungs, liver, and spleen.

##### Clinical Signs and Mortality Rates

**Figure 4:**
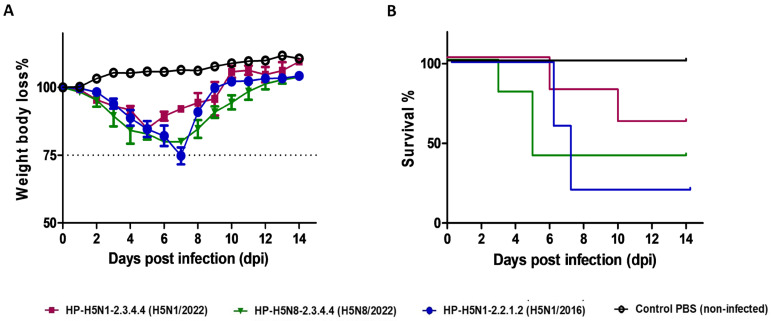
Virulence of highly pathogenic avian influenza strains: 24- to 26-week-old female C57BL/6 mice (*n* = 5) were mock-infected or intranasally inoculated with 100 µL of 10^6^ TCID50/mouse of H5N1/2022, H5N8/2022, and previously circulating H5N1/2016. Body weight loss (**A**) and survival (**B**) of mice were monitored for 14 days after viral infection. Body weight changes were monitored daily and expressed as percentage of initial weight. Values represent mean ± SEM (*n* = 5 per group at start). Data shown only for surviving mice at each time point.

Following intranasal inoculation, the three virus strains showed distinct patterns of morbidity and mortality in mice (Figure 4A,B). Body weight changes and survival were monitored daily for 14 days post-infection.

G1 H5N1/2022 demonstrated moderate clinical manifestations, with progressive weight loss reaching 15.4 ± 1.6% of initial body weight by day 5 (Figure 4A) while the lowest mortality rate: 40% by day 14 (Figure 4B) and with median survival time: 12 days. Neurological signs appeared in 20% (1/5) of mice, characterized by a mild hunched posture and reduced activity.

G2 H5N8/2022 showed intermediate pathogenicity. Maximum weight loss: 20.1 ± 1.8% by day 6 (Figure 4A), while mortality rate: 60% by day 14 (Figure 4B), with median survival time: 10 days. Moderate neurological signs, including an unstable gait and reduced responsiveness, were observed in 40% (2/5) of mice by day 5 post-infection.

The G3 H5N1/2016 strain induced the most severe disease clinical progression, with a dramatic weight loss of 25.3 ± 2.1% by day 7 (Figure 4A), while the highest mortality rate: 80% by day 14 (Figure 4B), with a median survival time: 8.5 days. Severe neurological manifestations, including paralysis and seizures, developed in 60% (3/5) of mice between days 4 and 6 post-infection.

The control group (G4) maintained a normal weight gain and behavior throughout the experimental period, with no mortality or clinical signs observed (Figure 4A,B). Weight loss patterns differed significantly between groups (Welch’s ANOVA, F = 15.23, *p* < 0.01). The log-rank test revealed significant differences in survival curves (χ^2^ = 18.45, *p* < 0.01), with H5N1/2016 showing a significantly higher mortality compared to the 2022 strains between all infected groups compared to the control (*p* < 0.05), with H5N1/2016 showing a significantly higher pathogenicity compared to both 2022 strains (*p* < 0.01).

##### Viral Replication in Mice Organs

**Figure 5:**
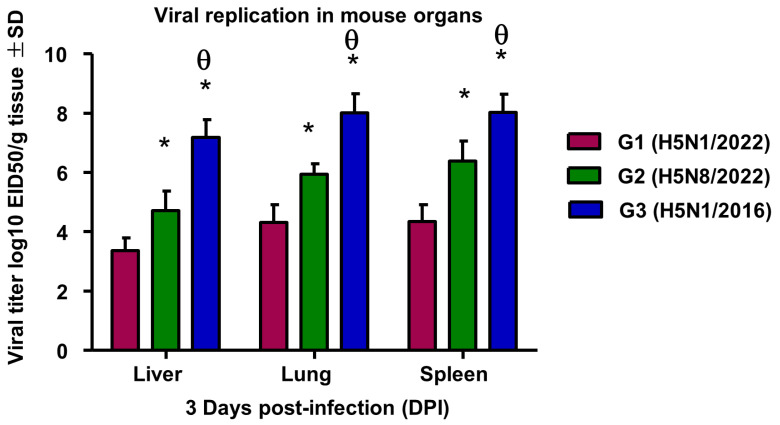
Viral replication in mice organs infected with different HPAIV strains. Values are presented as mean ± SD. *: statistically significant compared to corresponding value in group 1 (*p* < 0.05). θ: statistically significant compared to corresponding value in group 2 (*p* < 0.05). * *p* < 0.05 compared to H5N1/2016; *p* < 0.05 compared to H5N8/2022.

Analysis of tissue viral loads revealed strain-specific replication patterns (Figure 5). G3 (H5N1/2016) demonstrated the highest viral loads across all the examined organs (lung: 8.01 ± 0.64, liver: 7.19 ± 0.6, spleen: 8.03 ± 0.61 log10 EID50/g), aligning with its higher mortality rate in mice. G2 (H5N8/2022) showed intermediate replication levels (lung: 5.95 ± 0.35, liver: 4.71 ± 0.66, spleen: 6.38 ± 0.68 log10 EID50/g). G1 (H5N1/2022) exhibited the lowest viral loads (lung: 4.32 ± 0.59, liver: 3.36 ± 0.43, spleen: 4.35 ± 0.56 log10 EID50/g). A Kruskal–Wallis analysis with Dunn’s post hoc test revealed significant differences in viral loads between all groups (H = 16.78, *p* < 0.05).

##### Histopathological Findings in Mice

**Figure 6:**
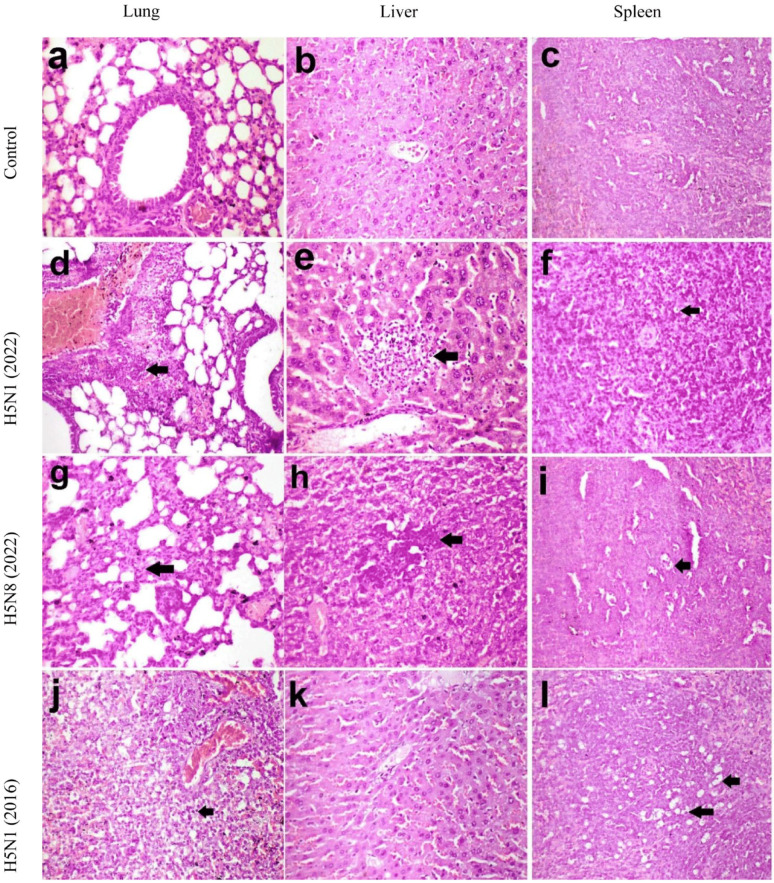
Histopathology of lungs, liver, and spleen of mice. (**a**) Normal histopathological alteration of lungs, (**b**) liver, and (**c**) spleen in control group, (**d**) perivascular leukocytic infiltration and hemorrhages in lungs, (**e**) leukocytic infiltration in liver, (**f**) lymphocytolysis in lymphoid follicles in spleen of mice in H5N1/2022 group, (**g**) interstitial pneumonia, (**h**) leukocytic infiltration in liver (**i**) lymphocytolysis in spleen of mice in H5N8/2022 group, (**j**) interstitial pneumonia, (**k**) mild histopathological alteration of the liver, and (**l**) lymphocytolysis in spleen of mice in H5N1/2016 group. Black arrows indicate pathological changes specific to each tissue type; in the lungs, they show areas of inflammatory cell infiltration and hemorrhage, while in the liver they show foci of leukocytic infiltration and in the spleen indicate regions of lymphoid depletion/lymphocytolysis. Hematoxylin and eosin stain X 200.

Microscopy of the lungs, liver, and spleen of mice in the control group revealed a normal histological structure (Figure 6a–c). Microscopy of the lungs of mice in the H5N1/2022 group revealed moderate perivascular leukocytic infiltration and hemorrhage, in addition to interstitial pneumonia (Figure 6d). The liver of mice in the H5N8/2022 group revealed leukocytic infiltration (Figure 6e), and the spleen had lymphocytolysis in lymphoid follicles (Figure 6f). In the H5N1/2016 group, the interstitial pneumonia, the leukocytic infiltration in the liver, and the lymphocytolysis in the spleen were more severe than in the previous group (Figure 6g–i). In the H5N8/2022 group, there was interstitial pneumonia and lymphocytolysis in the spleen (Figure 6j–l). Black arrows indicate pathological changes specific to each tissue type; in the lungs, they show areas of inflammatory cell infiltration and hemorrhage, while in the liver they show foci of leukocytic infiltration and in the spleen indicate regions of lymphoid depletion/lymphocytolysis.

Lesion scoring confirmed the highest severity in G3 across all the examined tissues, with significant differences between groups (*p* < 0.05). These histopathological findings correlated with the viral load measurements and clinical manifestations, demonstrating consistent strain-specific patterns of pathogenicity.

### 3.3. Correlation Between Viral Loads and Disease Severity

A statistical analysis of viral loads and disease severity was performed using Spearman’s rank correlation coefficient (r), with the significance determined at *p* < 0.001 using GraphPad Prism v9.0. In chickens, viral shedding levels correlated strongly with clinical severity scores (Spearman’s r = 0.83, *p* < 0.001). Peak viral shedding preceded mortality by 24–48 h, with birds showing the highest viral loads (>7.0 log10 EID50/mL) succumbing to infection within 2 days post-peak shedding. H5N8/2022 demonstrated the highest correlation between shedding and clinical scores (*r* = 0.89, *p* < 0.001), followed by H5N1/2022 (*r* = 0.81, *p* < 0.001) and H5N1/2016 (*r* = 0.78, *p* < 0.001).

In mice, tissue viral loads showed organ-specific correlations with disease severity. Lung viral loads strongly correlated with weight loss (r = 0.87, *p* < 0.001) and clinical scores (r = 0.85, *p* < 0.001). This correlation was most pronounced in the H5N1/2016 group, where lung viral loads >7.0 log10 EID50/g corresponded with severe clinical manifestations and mortality. Neurological symptoms showed a significant correlation with spleen viral loads (r = 0.79, *p* < 0.001), particularly in H5N1/2016-infected mice.

These correlations demonstrate direct relationships between viral replication efficiency and pathological outcomes, supporting strain-specific variations in virulence.

## 4. Discussion

The emergence and development of highly pathogenic avian influenza viruses represent major challenges to both animal and public health. An extensive analysis of the pathobiological characteristics of the recent and classical HPAI strains circulating in Egypt reveals key changes in traits concerning their virulence and patterns of transmission and host adaptation.

In the avian model, all three virus strains demonstrated a high pathogenicity, resulting in 100% mortality following intranasal inoculation. However, significant differences emerged in viral shedding patterns and transmission dynamics. The H5N8/2022 strain exhibited significantly higher viral shedding (Mann–Whitney U test, *p* < 0.05, effect size r = 0.76) (8.34 ± 0.55 log10 EID50) compared to both H5N1 strains, suggesting enhanced transmission potential [38]. That may be correlated with epidemiological data showing a fast geographical spread of H5N8/2022 viruses on more than one continent [39,40].

Higher viral shedding might be expected to enhance the efficient spread of this strain; however, it is important to emphasize that its contact transmission rate was 50%, compared with the 100% rate recorded for the classical H5N1/2016 strain. Such a trend, if unexpected, might indicate that recent evolutionary changes could have changed the dynamics of viral transmission; this adaptation pattern aligns with the strain’s enhanced replication in respiratory tissues but its modified transmissibility possibly due to a mutation affecting receptor binding specificity [41,42]. These differences in transmissibility may impact the potential for dissemination and establishment of viruses within poultry flocks [20].

An histopathological examination revealed strain-specific tissue damage patterns, with H5N1/2022 causing the most severe lesions in chicken respiratory tissues. This enhanced tissue tropism could reflect viral adaptations to the avian respiratory tract, potentially contributing to increased pathogenicity [43,44]. The severe tracheal damage observed correlates with higher viral loads in these tissues, suggesting efficient local replication.

A striking contrast emerged in the mammalian model, where the historical H5N1/2016 strain showed the highest pathogenicity (80% mortality) compared to the 2022 isolates (40–60% mortality). This differential virulence pattern might indicate evolutionary trade-offs during adaptation to avian hosts [45]. The reduced mammalian pathogenicity of newer strains could result from genetic changes affecting host range determinants, particularly in the viral polymerase complex [26,46,47].

Viral replication kinetics in mouse organs further supported this hypothesis, with H5N1/2016 showing significantly higher titers across all the tissues examined. This broad tissue tropism in mammals aligned with severe histopathological changes and clinical manifestations. The differential neurological manifestations observed in mice provide crucial insights into strain-specific pathogenic mechanisms. While H5N1/2016 induced severe neurological symptoms in 60% of mice, including paralysis and seizures, newer strains showed reduced neurotropism, with only mild to moderate manifestations (20–40% of mice) [26,48,49]. This reduction in neurological impact parallels the overall decrease in the mammalian pathogenicity of newer strains, suggesting coordinated changes in viral proteins affecting both their systemic spread and neuronal invasion. These findings have particular relevance for zoonotic risk assessment, as neurological complications represent a serious concern in mammalian infections [50].

The emergence of clade 2.3.4.4b viruses represents a significant evolutionary shift in the Egyptian HPAI ecology. Our data indicate these viruses have evolved toward enhanced replication in avian hosts, while showing reduced mammalian pathogenicity. This adaptation pattern differs from historical observations of H5N1 evolution in Egypt, where increased mammalian adaptation was often noted [51,52].

The complex evolution of HPAI viruses in Egypt continues to challenge control efforts. Our findings demonstrate that newly emerged H5Nx viruses maintain high pathogenicity in avian hosts, while showing variable adaptation to mammals. This evolving landscape requires continued vigilance and adaptive control strategies to manage both the veterinary and public health risks [53,54].

These findings have important implications for surveillance and control strategies. First, the higher viral shedding of H5N8/2022 necessitates enhanced biosecurity measures in poultry facilities. Second, the modified transmission patterns suggest a need to reevaluate current control protocols. Third, the reduced but persistent mammalian pathogenicity warrants continued monitoring for zoonotic potential.

The study limitations include the restricted number of strains analyzed and the use of a mouse model for mammalian pathogenicity, which may not fully reflect responses in other mammalian species. Additionally, a detailed genetic analysis of adaptive mutations was beyond our current scope but would provide valuable mechanistic insights. Future studies should consider including contact transmission assessment in mammalian models, particularly given the evolving nature of these viruses and their potential for enhanced mammalian adaptation

## 5. Conclusions

This study has shown major evolutionary changes in HPAI viruses circulating in Egypt. Contemporary strains show increased viral shedding and avian tissue tropism but decreased mammalian pathogenicity compared to historical isolates. The H5N8/2022 strain best exemplifies such an evolution, displaying increased viral shedding but decreased contact transmission efficiency. Such a study clearly exposes the intricate evolutionary route to improved avian adaptation, with the retention of mammalian infectivity, and it increases the need for continued surveillance with adaptive control strategies. These findings illustrate unique characteristics of virulence and transmission in avian versus mammalian hosts, a fact that makes it imperative to include host-specific factors in risk assessment and when formulating disease control strategies.

## Figures and Tables

**Table 1:** Virus strains used in this study.

Designation	Strain	Subtype	Clade	Origin
H5N1/2016	A/chicken/Egypt/S52/2016	H5N1	2.2.1.2	* RLVQCP
H5N1/2022	A/chicken/Egypt/F71-F114C/2022	H5N1	2.3.4.4b	Current surveillance
H5N8/2022	A/chicken/Egypt/F71-S86C/2022	H5N8	2.3.4.4b	Current surveillance

* RLVQCP: Reference Laboratory for Veterinary Quality Control on Poultry Production, Animal Health Research Institute (AHRI), Dokki, Giza, Egypt.

**Table 2:** Daily mortality count and cumulative mortality rate in infected and contact chickens with different HPAI virus strains.

Groups	Doses	Viruses	3 dpi	4 dpi	5 dpi	6 dpi	7 dpi	Mortality Rate%	Contact Transmission Rate (%)
G1	10^6^	H5N1/2022	3	4	1	1	1	100%	
G1\	contact	H5N1/2022				2			50%
G2	10^6^	H5N8/2022	2	6	2			100%	
G2\	contact	H5N8/2022				2			50%
G3	10^6^	H5N1/2016	3	6			1	100%	
G3\	contact	H5N1/2016		2			2		100%
G4	Neg. control	PBS						0%	

G1–G4: Experimental groups of directly inoculated chickens (*n* = 10 in each group). G1\–G3\: Contact exposure groups (*n* = 4 in each group). Numbers in 3–7 dpi columns represent chickens that died on that day.

## Data Availability

The original contributions presented in this study are included in the article. Further inquiries can be directed to the corresponding author.

## References

[1] Fusaro A., Gonzales J.L., Kuiken T., Mirinavičiūtė G., Niqueux É., Ståhl K., Staubach C., European Food Safety Authority, European Centre for Disease Prevention and Control, European Union Reference Laboratory for Avian Influenza (2024). Avian influenza overview December 2023–March 2024. EFSA J..

[2] Cui J., Qu N., Guo Y., Cao L., Wu S., Mei K., Sun H., Lu Y., Qin Z., Jiao P. (2017). Phylogeny, pathogenicity, and transmission of H5N1 avian influenza viruses in chickens. Front. Cell. Infect. Microbiol..

[3] Huang P., Sun L., Li J., Wu Q., Rezaei N., Jiang S., Pan C. (2023). Potential cross-species transmission of highly pathogenic avian influenza H5 subtype (HPAI H5) viruses to humans calls for the development of H5-specific and universal influenza vaccines. Cell Discov..

[4] Meseko C., Milani A., Inuwa B., Chinyere C., Shittu I., Ahmed J., Giussani E., Palumbo E., Zecchin B., Bonfante F. (2023). The Evolution of Highly Pathogenic Avian Influenza A (H5) in Poultry in Nigeria, 2021–2022. Viruses.

[5] Hassan K.E., El-Kady M.F., EL-Sawah A.A., Luttermann C., Parvin R., Shany S., Beer M., Harder T. (2021). Respiratory disease due to mixed viral infections in poultry flocks in Egypt between 2017 and 2018: Upsurge of highly pathogenic avian influenza virus subtype H5N8 since 2018. Transbound. Emerg. Dis..

[6] Salaheldin A.H., Kasbohm E., El-Naggar H., Ulrich R., Scheibner D., Gischke M., Hassan M.K., Arafa A.-S.A., Hassan W.M., Abd El-Hamid H.S. (2018). Potential biological and climatic factors that influence the incidence and persistence of highly pathogenic H5N1 avian influenza virus in Egypt. Front. Microbiol..

[7] Yehia N., Naguib M.M., Li R., Hagag N., El-Husseiny M., Mosaad Z., Nour A., Rabea N., Hasan W.M., Hassan M.K. (2018). Multiple introductions of reassorted highly pathogenic avian influenza viruses (H5N8) clade 2.3. 4.4 b causing outbreaks in wild birds and poultry in Egypt. Infect. Genet. Evol..

[8] El-Shesheny R., Moatasim Y., Mahmoud S.H., Song Y., El Taweel A., Gomaa M., Kamel M.N., Sayes M.E., Kandeil A., Lam T.T. (2023). Highly pathogenic avian influenza a (H5N1) virus clade 2.3. 4.4 b in wild birds and live bird markets, Egypt. Pathogens.

[9] Anis A., AboElkhair M., Ibrahim M. (2018). Characterization of highly pathogenic avian influenza H5N8 virus from Egyptian domestic waterfowl in 2017. Avian Pathol..

[10] Adlhoch C., Fusaro A., Gonzales J.L., Kuiken T., Marangon S., Niqueux É., Staubach C., European Food Safety Authority, European Centre for Disease Prevention and Control, European Union Reference Laboratory for Avian Influenza (2022). Avian influenza overview June–September 2022. EFSA J..

[11] Engelsma M., Heutink R., Harders F., Germeraad E.A., Beerens N. (2022). Multiple introductions of reassorted highly pathogenic avian influenza H5Nx viruses clade 2.3. 4.4 b causing outbreaks in wild birds and poultry in The Netherlands, 2020–2021. Microbiol. Spectr..

[12] Bakeer A.M., Khattab M.S., Aly M.M., Arafa A.-S., Amer F., Hafez H.M., Afify M.M. (2019). Estimation of Pathological and Molecular Findings in Vaccinated and Non-Vaccinated Chickens Challenged with Highly Pathogenic Avian Influenza H5N1 Virus. Pak. Vet. J..

[13] Kaplan E.L., Meier P. (1958). Nonparametric estimation from incomplete observations. J. Am. Stat. Assoc..

[14] Chan Y. (2003). Biostatistics 102: Quantitative data–parametric & non-parametric tests. Blood Press.

[15] Chen W., He B., Li C., Zhang X., Wu W., Yin X., Fan B., Fan X., Wang J. (2007). Real-time RT-PCR for H5N1 avian influenza A virus detection. J. Med. Microbiol..

[16] Lee D.-H., Criado M.F., Swayne D.E. (2021). Pathobiological origins and evolutionary history of highly pathogenic avian influenza viruses. Cold Spring Harb. Perspect. Med..

[17] Rohaim M.A., El Naggar R.F., Madbouly Y., AbdelSabour M.A., Ahmed K.A., Munir M. (2021). Comparative infectivity and transmissibility studies of wild-bird and chicken-origin highly pathogenic avian influenza viruses H5N8 in chickens. Comp. Immunol. Microbiol. Infect. Dis..

[18] Miao X., Feng M., Zhu O., Yang F., Yin Y., Yin Y., Chen S., Qin T., Peng D., Liu X. (2022). H5N8 Subtype avian influenza virus isolated from migratory birds emerging in Eastern China possessed a high pathogenicity in mammals. Transbound. Emerg. Dis..

[19] Baek Y.-G., Lee Y.-N., Lee D.-H., Shin J.-I., Lee J.-H., Chung D.H., Lee E.-K., Heo G.-B., Sagong M., Kye S.-J. (2021). Multiple reassortants of H5N8 clade 2.3. 4.4 b highly pathogenic avian influenza viruses detected in South Korea during the winter of 2020–2021. Viruses.

[20] Kwon J.H., Bahl J., Swayne D.E., Lee Y.N., Lee Y.J., Song C.S., Lee D.H. (2020). Domestic ducks play a major role in the maintenance and spread of H5N8 highly pathogenic avian influenza viruses in South Korea. Transbound. Emerg. Dis..

[21] Augustin T.T. (2021). Studies on the Control of Avian Influenza and Newcastle Disease. Ph.D. Thesis.

[22] Hao X., Hu J., Wang J., Xu J., Cheng H., Xu Y., Li Q., He D., Liu X., Wang X. (2016). Reassortant H5N1 avian influenza viruses containing PA or NP gene from an H9N2 virus significantly increase the pathogenicity in mice. Vet. Microbiol..

[23] Cattoli G., Milani A., Temperton N., Zecchin B., Buratin A., Molesti E., Aly M.M., Arafa A., Capua I. (2011). Antigenic drift in H5N1 avian influenza virus in poultry is driven by mutations in major antigenic sites of the hemagglutinin molecule analogous to those for human influenza virus. J. Virol..

[24] Liang Y., Nissen J.N., Krog J.S., Breum S.Ø., Trebbien R., Larsen L.E., Hjulsager C.K. (2021). Novel clade 2.3. 4.4 b highly pathogenic avian influenza A H5N8 and H5N5 viruses in Denmark, 2020. Viruses.

[25] Lycett S.J., Duchatel F., Digard P. (2019). A brief history of bird flu. Philos. Trans. R. Soc. B.

[26] Eisfeld A.J., Biswas A., Guan L., Gu C., Maemura T., Trifkovic S., Wang T., Babujee L., Dahn R., Halfmann P.J. (2024). Pathogenicity and transmissibility of bovine H5N1 influenza virus. Nature.

[27] Russell C.A., Fonville J.M., Brown A.E., Burke D.F., Smith D.L., James S.L., Herfst S., Van Boheemen S., Linster M., Schrauwen E.J. (2012). The potential for respiratory droplet–transmissible A/H5N1 influenza virus to evolve in a mammalian host. Science.

[28] Verhagen J.H., Herfst S., Fouchier R.A. (2015). How a virus travels the world. Science.

[29] Reed L.J., Muench H. (1938). A simple method of estimating fifty per cent endpoints. Am. J. Epidemiol..

[30] IBM Corp (2013). Statistics I. Released 2013. IBM SPSS Statistics for Windows.

[31] Ghasemi A., Zahediasl S. (2012). Normality tests for statistical analysis: A guide for non-statisticians. Int. J. Endocrinol. Metab..

[32] Kim T.K. (2017). Understanding one-way ANOVA using conceptual figures. Korean J. Anesthesiol..

[33] McKnight P.E., Najab J. (2010). Mann-Whitney U Test. The Corsini Encyclopedia of Psychology.

[34] Moder K. (2010). Alternatives to F-test in one way ANOVA in case of heterogeneity of variances (a simulation study). Psychol. Test Assess. Model..

[35] Schober P., Boer C., Schwarte L.A. (2018). Correlation coefficients: Appropriate use and interpretation. Anesth. Analg..

[36] Lang T.A., Altman D.G. (2015). Basic statistical reporting for articles published in biomedical journals: The “Statistical Analyses and Methods in the Published Literature” or the SAMPL Guidelines. Int. J. Nurs. Stud..

[37] Percie du Sert N., Hurst V., Ahluwalia A., Alam S., Avey M.T., Baker M., Browne W.J., Clark A., Cuthill I.C., Dirnagl U. (2020). The ARRIVE guidelines 2.0: Updated guidelines for reporting animal research. J. Cereb. Blood Flow Metab..

[38] Ramey A.M., Reeves A.B. (2020). Ecology of influenza A viruses in wild birds and wetlands of Alaska. Avian Dis..

[39] Saad N., Esaki M., Kojima I., Khalil A.M., Osuga S., Shahein M.A., Okuya K., Ozawa M., Alhatlani B.Y. (2024). Phylogenetic Characterization of Novel Reassortant 2.3. 4.4 b H5N8 Highly Pathogenic Avian Influenza Viruses Isolated from Domestic Ducks in Egypt During the Winter Season 2021–2022. Viruses.

[40] Takadate Y., Tsunekuni R., Kumagai A., Mine J., Kikutani Y., Sakuma S., Miyazawa K., Uchida Y. (2023). Different infectivity and transmissibility of H5N8 and H5N1 high pathogenicity avian influenza viruses isolated from chickens in Japan in the 2021/2022 season. Viruses.

[41] Islam A., Ara T., Amin E., Islam S., Sayeed M.A., Shirin T., Hassan M.M., Klaassen M., Epstein J.H. (2023). Epidemiology and evolutionary dynamics of high pathogenicity avian influenza (HPAI) H5N1 in Bangladesh. Transbound. Emerg. Dis..

[42] Scheibner D., Salaheldin A.H., Bagato O., Zaeck L.M., Mostafa A., Blohm U., Müller C., Eweas A.F., Franzke K., Karger A. (2023). Phenotypic effects of mutations observed in the neuraminidase of human origin H5N1 influenza A viruses. PLoS Pathog..

[43] Seekings A.H., Warren C.J., Thomas S.S., Lean F.Z., Selden D., Mollett B.C., van Diemen P.M., Banyard A.C., Slomka M.J. (2023). Different outcomes of chicken infection with UK-origin H5N1-2020 and H5N8-2020 high-pathogenicity avian influenza viruses (clade 2.3. 4.4 b). Viruses.

[44] Dai M., Zhu S., An Z., You B., Li Z., Yao Y., Nair V., Liao M. (2023). Dissection of key factors correlating with H5N1 avian influenza virus driven inflammatory lung injury of chicken identified by single-cell analysis. PLoS Pathog..

[45] Kim I.-H., Nam J.-H., Kim C.-K., Choi Y.J., Lee H., An B.M., Lee N.-J., Jeong H., Lee S.-Y., Yeo S.-G. (2024). Pathogenicity of Highly Pathogenic Avian Influenza A (H5N1) Viruses Isolated from Cats in Mice and Ferrets, South Korea, 2023. Emerg. Infect. Dis..

[46] Taft A.S., Ozawa M., Fitch A., Depasse J.V., Halfmann P.J., Hill-Batorski L., Hatta M., Friedrich T.C., Lopes T.J., Maher E.A. (2015). Identification of mammalian-adapting mutations in the polymerase complex of an avian H5N1 influenza virus. Nat. Commun..

[47] Salaheldin A.H., Veits J., Abd El-Hamid H.S., Harder T.C., Devrishov D., Mettenleiter T.C., Hafez H.M., Abdelwhab E.M. (2017). Isolation and genetic characterization of a novel 2.2. 1.2 a H5N1 virus from a vaccinated meat-turkeys flock in Egypt. Virol. J..

[48] Chothe S.K., Srinivas S., Misra S., Nallipogu N.C., Gilbride E., LaBella L., Mukherjee S., Gauthier C.H., Pecoraro H.L., Webb B.T. (2024). Marked neurotropism and potential adaptation of H5N1 Clade 2.3. 4.4. b virus in naturally infected domestic cats. Emerg. Microbes Infect..

[49] Pulit-Penaloza J.A., Brock N., Belser J.A., Sun X., Pappas C., Kieran T.J., Basu Thakur P., Zeng H., Cui D., Frederick J. (2024). Highly pathogenic avian influenza A (H5N1) virus of clade 2.3. 4.4 b isolated from a human case in Chile causes fatal disease and transmits between co-housed ferrets. Emerg. Microbes Infect..

[50] Elsmo E.J., Wünschmann A., Beckmen K.B., Broughton-Neiswanger L.E., Buckles E.L., Ellis J., Fitzgerald S.D., Gerlach R., Hawkins S., Ip H.S. (2023). Highly pathogenic avian influenza A (H5N1) virus clade 2.3. 4.4 b infections in wild terrestrial mammals, United States, 2022. Emerg. Infect. Dis..

[51] Graziosi G., Lupini C., Catelli E., Carnaccini S. (2024). Highly pathogenic avian influenza (HPAI) H5 clade 2.3. 4.4 b virus infection in birds and mammals. Animals.

[52] Fusaro A., Zecchin B., Giussani E., Palumbo E., Agüero-García M., Bachofen C., Bálint Á., Banihashem F., Banyard A.C., Beerens N. (2024). High pathogenic avian influenza A (H5) viruses of clade 2.3. 4.4 b in Europe—Why trends of virus evolution are more difficult to predict. Virus Evol..

[53] Arafa A.-S., Yamada S., Imai M., Watanabe T., Yamayoshi S., Iwatsuki-Horimoto K., Kiso M., Sakai-Tagawa Y., Ito M., Imamura T. (2016). Risk assessment of recent Egyptian H5N1 influenza viruses. Sci. Rep..

[54] Peacock T., Moncla L., Dudas G., VanInsberghe D., Sukhova K., Lloyd-Smith J.O., Worobey M., Lowen A.C., Nelson M.I. (2025). The global H5N1 influenza panzootic in mammals. Nature.

